# Shaping the landscape of the *Escherichia coli* chromosome: replication-transcription encounters in cells with an ectopic replication origin

**DOI:** 10.1093/nar/gkv704

**Published:** 2015-07-08

**Authors:** Darja Ivanova, Toni Taylor, Sarah L. Smith, Juachi U. Dimude, Amy L. Upton, Mana M. Mehrjouy, Ole Skovgaard, David J. Sherratt, Renata Retkute, Christian J. Rudolph

**Affiliations:** 1 Division of Biosciences, College of Health and Life Sciences, Brunel University London, Uxbridge UB8 3PH, UK; 2 Department of Biochemistry, University of Oxford, South Parks Road, Oxford OX1 3QU, UK; 3 The Faculty of Health Sciences, Department of Cellular and Molecular Medicine, Copenhagen University, 2200 Copenhagen N, Denmark; 4 Department of Science, Systems and Models, Roskilde University, DK-4000 Roskilde, Denmark; 5 School of Veterinary Medicine & Science, University of Nottingham, Sutton Bonington Campus, Loughborough LE12 5RD, UK

## Abstract

Each cell division requires the unwinding of millions of DNA base pairs to allow chromosome duplication and gene transcription. As DNA replication and transcription share the same template, conflicts between both processes are unavoidable and head-on collisions are thought to be particularly problematic. Surprisingly, a recent study reported unperturbed cell cycle progression in *Escherichia coli* cells with an ectopic replication origin in which highly transcribed *rrn* operons were forced to be replicated opposite to normal. In this study we have re-generated a similar strain and found the doubling time to be twice that of normal cells. Replication profiles of this background revealed significant deviations in comparison to wild-type profiles, particularly in highly transcribed regions and the termination area. These deviations were alleviated by mutations that either inactivate the termination area or destabilise RNA polymerase complexes and allow their easier displacement by replication forks. Our data demonstrate that head-on replication-transcription conflicts are highly problematic. Indeed, analysis of the replication profile of the previously published *E. coli* construct revealed a chromosomal rearrangement that alleviates replication-transcription conflicts in an intriguingly simple way. Our data support the idea that avoiding head-on collisions has significantly contributed to shaping the distinct architecture of bacterial chromosomes.

## INTRODUCTION

**Figure 1.**
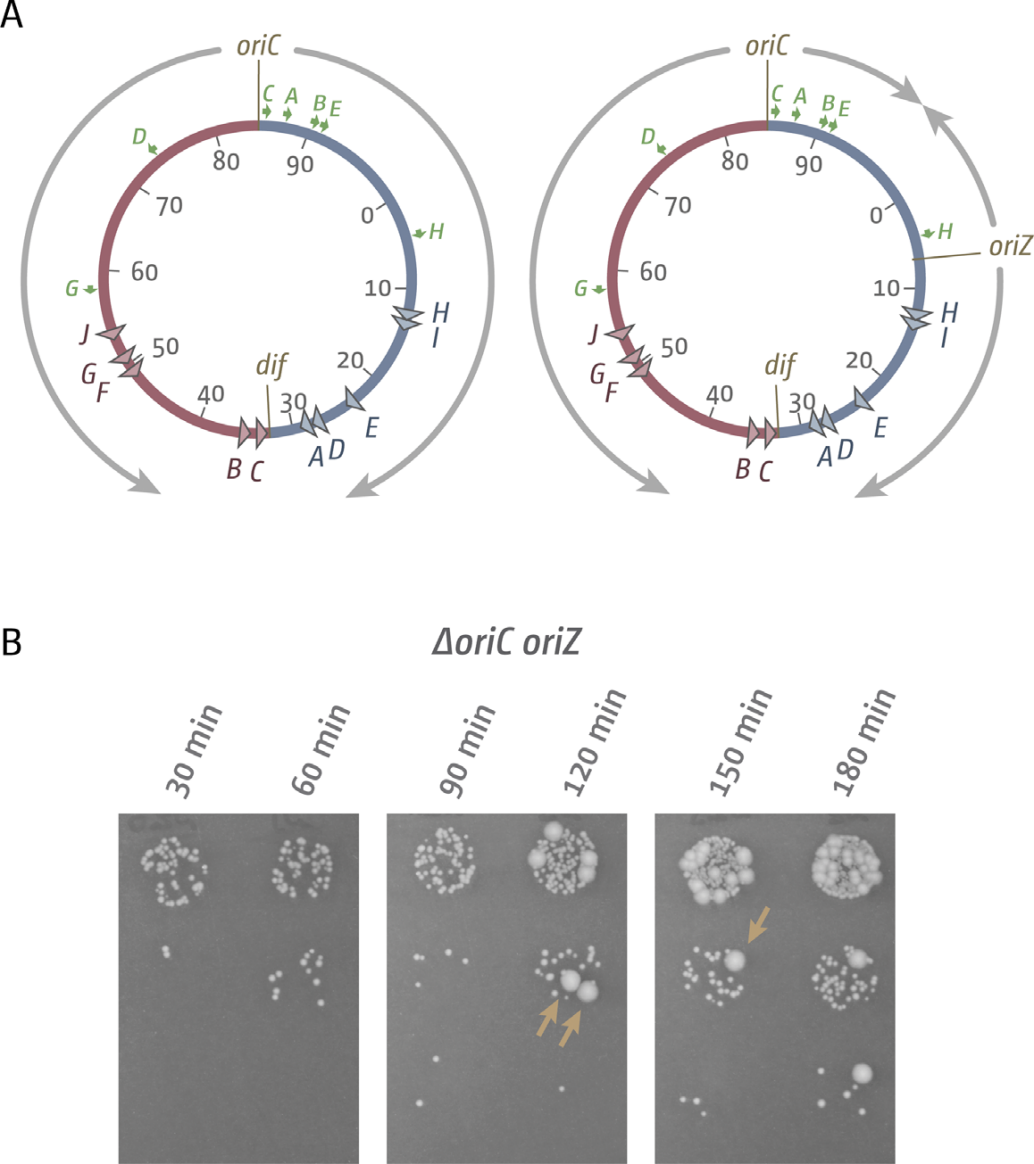
Chromosome replication and cell growth in cells with one or two replication origins. (**A**) Schematic representation of the replichore arrangement of *E. coli* chromosomes with one and two replication origins. *oriZ* indicates the integration of a duplication of the *oriC* sequence near the *lacZYA* operon (26). Direction of replication from the origins is indicated by gray arrows. The *dif* chromosome dimer resolution site is indicated. *ter* sites are highlighted by triangles and identified by their corresponding letter (‘A’ indicates the *terA* site). The numbers represent the minutes of the standard genetic map (0–100 min). Green arrows represent location and direction of transcription of the 7 *rrn* operons A–E, G and H. (**B**) Evaluation of the viable titre of a *ΔoriC oriZ* construct via spot dilutions of a growing culture at the times indicated. Large colony variants carrying suppressor mutations are highlighted by brown arrows. The strain used was RCe578 (*ΔoriC oriZ*).

The accurate and processive replication of the genetic material and the coordinated segregation of the two generated chromosomes into daughter cells is one of the most fundamental tasks of a cell to ensure preservation of genomic integrity and the maintenance of cell viability. In *Escherichia coli*, replication and segregation of the circular chromosome as well as cell division are highly coordinated events (1,2). Initiation of chromosomal replication is strictly regulated by the DnaA initiator protein, which coordinates recruitment of the replication machinery to a single replication origin, *oriC* (3,4). Two forks are established and move in opposite directions with a very high speed of up to 1000 nt/s until they fuse within a specialised termination zone opposite the origin (Figure 1A) (2,5). This zone is flanked by *ter* sequences (*terA–J*) that are bound by Tus protein, forming polar traps that restrict fork movement (6,7). These elements together distinctly define the architecture of bacterial chromosomes. The chromosome is divided into two replichores, one replicated by the fork moving clockwise, the other by the fork moving anticlockwise and the termination area actively prevents forks from entering the other replichore (1,6–7).

In rapidly growing cells substantial levels of gene expression are required. As transcription has the same template as DNA replication, but moves at a pace 10–20 × slower than replication (8,9), some degree of conflict between the two processes is unavoidable. Indeed, it was noted that within a single replichore, transcription of highly transcribed genes takes place preferentially on the leading strand template, which results in co-directional movement of replication and transcription (10). With the dawn of the post-genomic era bacterial sequence information could be analysed more systematically, revealing a strikingly high degree of co-directionality of replication and transcription especially of highly transcribed genes (11,12). In *E. coli* the overall co-directionality is only 54%, however, 93% of highly transcribed genes that code for ribosomal proteins are transcribed co-directionally with replication (10,11). In other bacteria, such as *Bacillus subtilis*, the general co-directionality is even higher (>70% overall) (11). In eukaryotic cells no overall bias of replication and transcription was found, suggesting that the orientation of open reading frames is effectively random. However, in yeast a replication barrier prevents forks from entering the highly transcribed ribosomal DNA repeats in a head-on orientation (12–14), suggesting that replication-transcription encounters might be problematic in transcriptionally very active areas of the chromosome. In contrast, replication fork fusion was found throughout ribosomal DNA repeats in human cells, suggesting that co-directionality is not established in human cells (15,16)

The co-directionality of replication and transcription, particularly if genes are highly transcribed, has led to the suggestion that head-on encounters of replisomes with elongating RNA polymerase complexes might be rather problematic, at least in bacteria and yeast (17–20). Experiments in *E. coli* strains with inverted *rrn* operons support this idea (14,21–23). These results are in line with global *in vivo* evidence in *B. subtilis* strains in which the chromosome is asymmetrically replicated from a single ectopic origin (24,25). A comparison of the resulting replication profiles revealed that replication-transcription encounters are problematic, especially if they occur in highly transcribed areas of the chromosome (24,25). In contrast, recent experiments in *E. coli* with a similarly shifted replication origin revealed a doubling time surprisingly close to wild-type cells, suggesting the situation might be different (26).

In this study, we have regenerated strains with two origins and with a single ectopic replication origin. Our data show clearly that *E. coli* cells with an ectopic replication origin have considerable difficulties, resulting in much elongated doubling times and deviations in their replication profiles in comparison to the replication profile of wild-type cells. By inactivating the replication fork trap in the termination zone we are able to show that the elongated doubling time is partially caused by the asymmetric replication pattern, with one fork being blocked after a quarter of the chromosome is replicated, while the other fork has to replicate the other three quarters. In addition, the replication profile shows deviations in regions where highly transcribed genes are replicated in head-on orientation, most notably at the *rrnCABE* operon cluster. A point mutation which reduces the ability of RNA polymerase transcription complexes to pause and backtrack (27), thereby reducing the effect of conflicts between replication and transcription (27,28), significantly reduces the doubling time and alleviates the deviations of the replication profile at the *rrnCABE* operon cluster. This confirms that head-on replication-transcription encounters in *E. coli* significantly interfere with replication fork progression, and that the most severe effects are observed in highly transcribed regions of the genome, in line with previous results (21–25). Thus, the fast doubling time of the previously reported *E. coli* strain with the ectopic replication origin (26) is likely to be caused by a compensatory mutation. We generated a replication profile of this particular construct and found indeed a genomic rearrangement which alleviates the above problems in an intriguingly simple way.

## MATERIALS AND METHODS

### Bacterial strains and general methods

For *E. coli* K12 strains see Table 1. Strains were either constructed via P1*vir* transduction (29), by single step gene disruptions (30) or as cited (26,31–34).

**Table 1.** *Escherichia coli* K-12 strains

Strain number	Relevant genotype^a^	Source
**AB1157 derivatives**		
WX296	*oriZ-cat-frt*	(26)
WX340	*ΔoriC-frt oriZ-cat-frt lac240-hyg[3908] tetO240-gen[365] ΔgalK::Plac-tetR-mcerulean-frt ΔleuB::Plac-lacI-mcherry-frt*	(26)
**MG1655 derivatives**		
MG1655	F^−^*rph-1*	(31)
N4849	rpoB*35	(32)
N5925	rpo*35 ΔlacIZYA	(33)
N6796	*tus1::dhfr*	(41)
N8227	*Δtus::cat*	(41)
RCe395	rpoB*35 tnaA::Tn*10 dnaA46 rnhA::cat tus1::dhfr ΔoriC::kan*^b^	(41)
RCe504	*oriZ-cat-frt*	MG1655 × P1.WX296 to Cm^r^
RCe544	*ΔlacIZYA oriZ-cat-frt*	TB28 × P1.WX296 to Cm^r^
RCe566	rpoB*35 oriZ-cat-frt	N4849 × P1.WX296 to Cm^r^
RCe567	*oriZ-cat-frt tus1::dhfr*	RCe504 × P1.N6706 to Tm^r^
RCe572	*oriZ-cat-frt tus1::dhfr ΔoriC::kan*^b^	RCe567 × P1.RCe395 to Km^r^
RCe573	rpoB*35 oriZ-cat-frt ΔoriC::kan^b^	RCe566 × P1.RCe395 to Km^r^
RCe574	rpoB*35 oriZ-cat-frt tus1::dhfr	RCe566 × P1.N6706 to Tm^r^
RCe576	rpoB*35 oriZ-cat-frt tus1::dhfr ΔoriC::kan^b^	RCe574 × P1.RCe395 to Km^r^
RCe578	*ΔoriC::kan*^b^*oriZ-cat-frt*	RCe504 × P1.RCe395 to Km^r^
TB28	*ΔlacIZYA<>frt*	(34)

^a^Only the relevant additional genotype of the derivatives is shown. The abbreviations *kan*, *cat*, *dhfr*, *gen* and *hyg* refer to insertions conferring resistance to kanamycin (Km^r^), chloramphenicol (Cm^r^), trimethoprim (Tm^r^), gentamicin (Gm^r^) and hygromycin (Hyg^r^), respectively. Tn*10* confers resistance to tetracycline (Tc^r^). *frt* stands for the 34 bp recognition site of the FLP/*frt* site-directed recombination system.

^b^*ΔoriC* refers to a replacement of the entire origin region (754 bp) including DnaA boxes and 13mers as well as the entire *mioC* gene by a kanamycin resistance cassette (41).

### Growth media

Luria broth (LB) and agar was modified from (35) as follows: 1% tryptone (Bacto™, BD Biosciences), 0.5% yeast extract (Bacto™, BD Biosciences) and 0.05% NaCl (Sigma–Aldrich). Glucose was omitted and the pH adjusted to 7.4. Minimal medium ‘56’ was prepared according to (36): 74 mM KH_2_PO_4_, 120 mM Na_2_HPO_4_, 3.4 mM MgSO_4_·7H_2_O, 30 mM (NH)_2_SO_4_, 85 μM Ca(NO_3_)_2_ and 3.6 μM FeSO_4_·7H_2_O. The pH was adjusted to 7.4. This was used at one-half strength, denoted 56/2.

### Marker frequency analysis by deep sequencing

Samples from cultures of a strain grown over night in LB broth were diluted 100-fold in fresh broth and incubated with vigorous aeration until an *A*_600_ reached 0.4 at the temperature indicated. The only exception was the *ΔoriC oriZ* background, for which growth was initiated from a single colony from a streak plate (see Supplementary Figure S1A) to avoid suppressors formed in the overnight culture outgrowing the slow growing *ΔoriC oriZ* cells. The culture was then diluted again 100-fold in pre-warmed fresh broth and grown again until an *A*_600_ of 0.4 was reached. Samples from these exponential phase cultures were flash-frozen in liquid nitrogen at this point for subsequent DNA extraction. Growth curves were recorded using the same procedure (see below), demonstrating that cultures grown to an *A*_600_ of 0.4 did not show any sign of transition into stationary phase. Incubation of the remaining culture was continued until several hours after the culture had saturated and showed no further increase in the *A*_600_. A further sample (stationary phase) was frozen at this point. DNA was then extracted using the GenElute Bacterial Genomic DNA Kit (Sigma-Aldrich). Marker frequency analysis was performed using Illumina HiSeq 2500 sequencing (fast run) to measure sequence copy number. Sequencing of the AB1157 *ΔoriC oriZ* construct was performed on an Ion Torrent Proton sequencer. Enrichment of uniquely mapping sequence tags, in 1 kb windows, was calculated for an exponentially growing (replicating) sample relative to a non-replicating stationary phase wild-type sample to correct for differences in read depth across the genome and to allow presentation of the data as a marker frequency, as described previously (37–39). All relevant raw sequencing data can be accessed at the European Nucleotide Archive (http://www.ebi.ac.uk/ena/data/view/PRJEB9476).

### LOESS regression

A LOESS regression allows for a simplified visualisation of complex data sets. For a LOESS regression relatively simple models are fitted to defined small subsets of data points in order to develop a function describing the deterministic part of the variation in the data. Weighted least squares are used to fit a low-degree polynomial to a specified percentage of the data points. Data points are weighted by a smooth decreasing function of their distance to the smoothed point, giving more weight to points closer to the point whose response is being estimated, while less weight is given to points further away. We used a second order polynomial for local fit, tricube as weight function and set a fraction of data used for smoothing to 10%, which corresponds to a smoothing window around 460 kbp (40). To account for circularity of the chromosome, we used periodic boundary conditions.

### Growth curves

Samples from cultures of a strain grown over night in LB broth were diluted 100-fold in fresh broth and incubated with vigorous aeration at 37°C until *A*_600_ reached 0.4. The only exception was the *ΔoriC oriZ* background, for which growth was initiated from a single colony from a streak plate (see Supplementary Figure S1A) to avoid suppressors formed in the overnight culture outgrowing the slow growing *ΔoriC oriZ* cells. Upon reaching an *A*_600_ of 0.4, the culture was diluted again 100-fold in pre-warmed fresh broth and grown under identical conditions. Samples were taken every 30 min, diluted in 56/2 (see above) to 10^−7^ and 10 μl aliquots of each dilution dropped onto LB agar plates. For each dilution series 2 sets of drops were spotted. Colonies were counted after incubation for 18–24 h at 37°C. Mean colony numbers from both dilution series were calculated and a growth curve plotted. A suitable period where growth was exponential was selected to calculate the doubling time (usually between 60 and 180 min following dilution into fresh LB).

## RESULTS

In previous experiments from the Grossman lab, it was demonstrated by DNA microarrays that replication fork progression in *B. subtilis* cells with an ectopic replication origin was asymmetric (25). Replication forks moving against the direction of transcription were significantly slowed, supporting the idea that the problems associated with replication-transcription encounters have generated a bias for the co-directionality of both processes (25). In contrast, recent results in *E. coli* with a similar chromosome replication set-up suggested otherwise (26). The Sherratt lab reported that the doubling time of an *E. coli* AB1157 construct with the *oriC* sequence moved from 3.92 to 0.344 Mbp (called *oriZ*), roughly 1 Mb away from its original location, was surprisingly similar to both wild-type cells and a strain with two origins (20) The replication period was elongated due to the fact that the counter-clockwise replichore is extended by ∼1 Mbp, but there was no indication of a delay of ongoing replication by the assays employed (20).

As this appeared to be in contrast to existing *B. subtilis* and *E. coli* data (21–22,24–25), we decided to re-generate an *E. coli* construct by moving *oriZ* into an MG1655 background to analyse replication parameters. We generated a double origin construct (Figure 1A) without difficulty and moved a *ΔoriC::kan* deletion into this background via P1*vir* transduction. This transduction resulted in a reasonable number of transductants, confirming that a *ΔoriC oriZ* construct can be generated without much difficulty. However, when streaked to single colonies, *ΔoriC oriZ* transductants showed a variation of colony sizes, indicative of a growth defect and the accumulation of suppressor mutations.

We measured doubling times for MG1655, *oriC^+^ oriZ* and *ΔoriC oriZ* constructs in LB broth. Both MG1655 and *oriC^+^ oriZ* showed a doubling time of ∼20 min (Table 2), as reported (26). In contrast, our *ΔoriC oriZ* construct showed a doubling time of at least 40 min (Table 2) and growth curves were obtained only with difficulties. If suppressor mutations were present in the initial culture, these outgrew the *ΔoriC oriZ* background, leading to a biphasic growth curve (Supplementary Figure S1A). Even if we cultured from a carefully selected small colony from the parent plate, the accumulation of suppressors was easily demonstrated in our growth experiments (Figure 1B). Thus, our data suggest that *ΔoriC oriZ* cells show a severe growth defect, as data from previous studies would predict (21–22,24–25). In addition, our data imply that the much shorter doubling time reported before (26) might have been caused by the rapid accumulation of suppressor mutations.

**Table 2.** Doubling times in *E. coli* strains with one and two replication origins

Strain background	Doubling time	SD
MG1655	19.9	±1.1
*oriC^+^ oriZ*	20.6	±1.3
*ΔoriC oriZ*	39.8	±2.8
*oriC^+^ oriZ tus*	21.5	±0.7
oriC^+^ oriZ rpoB*35	23.1	±0.6
oriC^+^ oriZ tus rpoB*35	24.5	±0.9
*ΔoriC oriZ tus*	29.2	±1.4
ΔoriC oriZ rpoB*35	32.0	±0.8
ΔoriC oriZ tus rpoB*35	29.8	±3.8
MG1655^a^	20.8	n.d.
rpoB*35^a^	25.7	n.d.

^a^See Supplementary Methods and Supplementary Figure S1B for additional information.

**Figure 2.**
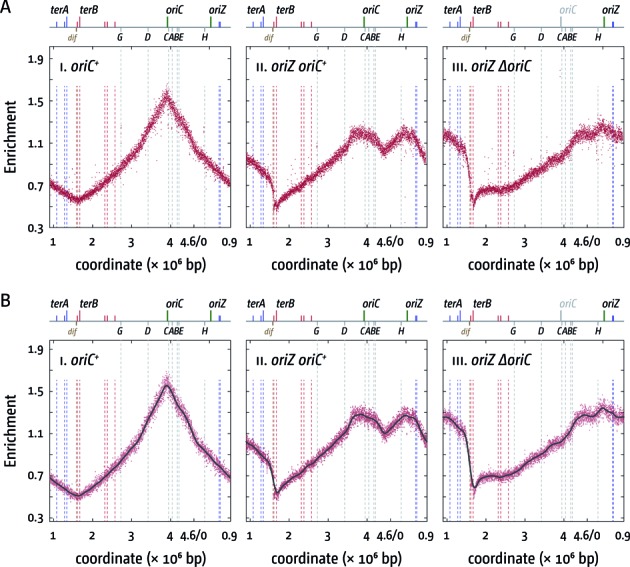
Replication profiles of *E. coli* cells with one and two replication origins. (**A**) Marker frequency analysis of *E. coli oriC^+^*, *oriC^+^ oriZ* and *ΔoriC oriZ* cells. The number of reads (normalized against reads for a stationary phase wild-type control) is plotted against the chromosomal location, starting at 0.9 Mbp for a better visualisation of both replication origins. A schematic representation of the *E. coli* chromosome showing positions of *oriC* and *oriZ* (green line; gray if deleted/not present) and *ter* sites (above) as well as *dif* and *rrn* operons A–E, G and H (below) is shown above the plotted data. The strains used were MG1655 (*oriC^+^*), RCe544 (*oriC^+^ oriZ*) and RCe578 (*ΔoriC oriZ*). (**B**) Panels i–iii show a LOESS regression curve (see Material & Methods) of the marker frequency data. As the LOESS regression is sensitive to outliers, we removed data points from the sets shown in (A) if they were beyond the threshold (measured enrichment − LOESS value)^2^ > 0.02.

**Figure 3.**
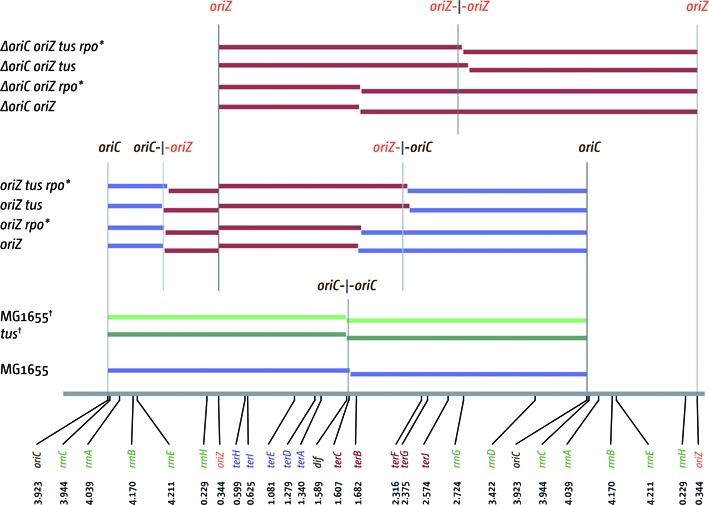
Replichore parameters of *E. coli* cells with one and two replication origins. A schematic of the *E. coli* chromosome is shown at the bottom, highlighting the location and coordinates of *oriC*, *oriZ*, the chromosome dimer resolution site *dif*, *ter* sites A–J and *rrn* operons A–E, G and H. The relevant genotypes are stated on the left, with colored bars representing the length of the replichore from each of the origins present, as calculated by the LOESS minima (see Table 2). Arithmetic mid-points between origin(s) are highlighted by gray lines. For MG1655 and *tus* marked with a dagger (†) the data sets were published before (41) and a LOESS regression curve was calculated to obtain termination points via LOESS minima.

We conducted marker frequency analyses (MFA) by deep sequencing to establish replication profiles in wild-type, *oriC^+^ oriZ* and *ΔoriC oriZ* backgrounds (Figure 2). The MFA is based on the ratio of uniquely mapped sequence reads in a replicating sample to a non-replicating control sample (stationary phase wild-type cells) sequenced in parallel. The data for our wild-type profile fits well with previous data sets (37–38,41). We used a LOESS regression curve (see ‘Materials and Methods’ section) to establish the minimum of the profile, which was located in close vicinity of the *terC* termination site (Table 3; Figure 3). As shown in Figure 3, the theoretical midpoint for both replichores is located at 1.603 Mbp, just between *dif* and *terC*. In the wild-type profile established for this study the low point was located at 1.629 Mbp, just beyond *terC* in between *terC* and *terB* (Table 3). This is in good agreement with previous data showing that a significant number of replication forks terminate at *terC* (42). However, previously we reported that replication profiles of wild type and *tus^−^* cells show surprisingly few differences (41). If forks terminate at *terC* because of the *terC*/Tus complex, a *tus^−^* strain might exhibit a shift of the termination point. As we did not establish a LOESS regression curve for these previous samples, we re-analysed our datasets for wild type and a *tus^−^* background (Supplementary Figure S2) to establish whether the deletion of *tus* causes any change in the curve minimum. As shown in Table 3, for the earlier datasets the termination point was located slightly closer to *dif*, between *dif* and *terC*. The calculated minima for wild type and *tus^−^* cells were identical (Table 3), suggesting that the absence of Tus protein does not influence the termination point of the two replisomes in the majority of cells.

**Table 3.** Replication profile minima established by the LOESS regression of the replication profile of *E. coli* strains with one and two replication origins

Strain background	Location of terminus-proximal LOESS minima (Mbp)	Location of *oriC–oriZ* LOESS minima (Mbp)	Arithmetic mid points (Mbp)
MG1655	1.629	n/a	1.603
MG1655 (41)	1.591	n/a	1.603
*tus* (41)	1.591	n/a	1.603
*oriC^+^ oriZ*	1.699	4.459	2.1335; 4.4535
*oriC^+^ oriZ tus*	2.199	4.449	2.1335; 4.4535
oriC^+^ oriZ rpoB*35	1.729	4.469	2.1335; 4.4535
oriC^+^ oriZ tus rpoB*35	2.179	4.499	2.1335; 4.4535
*ΔoriC oriZ tus*	2.769	n/a	2.664
ΔoriC oriZ rpoB*35	1.719	n/a	2.664
ΔoriC oriZ tus rpoB*35	2.709	n/a	2.664
*ΔoriC oriZ*	1.709	n/a	2.664

The profile of our MG1655 *oriC^+^ oriZ* construct was very similar to the previously established profile of an AB1157 *oriC^+^ oriZ* background (Figure 2) (41). The minimum between *oriC* and *oriZ* is located at 4.459 Mbp, which is close to the theoretical mid-point at 4.4535 Mbp (Figure 3; Table 3), but slightly shifted toward *oriZ*. As shown before (41) the termination area shows a distinct step in between *terA* and *terB/C*. Because forks coming from *oriZ* are able to reach this location much earlier than forks coming from *oriC* but then are blocked at *terC*/Tus or *terB*/Tus, on a population basis there will be significantly more cells that have replicated the area between *terA* and *terC/B* than the other side, resulting in the observed step of the profile. The termination area shows two low points, which coincide well with *terC* and *terB*. The calculated LOESS curve minimum is at 1.699 Mbp, which is in close proximity to *terB* (Figure 3). Thus, our data identify *terC* and *terB* as strong replication blocks. The LOESS minimum at 1.699 Mbp suggests a shift of the termination point towards *terB*, which is likely to be caused by the asymmetric replichore arrangement. However, forks still terminate at either *terC* or *terB*, with little evidence of forks progressing into the opposite replichore (Figures 2 and 3).

The replication profile of *ΔoriC oriZ* cells shows the clear absence of *oriC* activity, leading to an even more pronounced asymmetry, as forks still terminate at either *terC* or *terB* (Figure 2iii). In addition, there are some deviations of the *ΔoriC oriZ* replication profile from the profile observed in wild-type cells, with the two most noticeable being located around 4.2 and 0.23 Mbp (Figure 2iii). These deviations coincide with the location of the *rrnH* operon (0.229 Mbp) as well as the *rrnCABE* cluster (3.94–4.21 Mbp). Replication coming from *oriZ* will progress into these areas in the wrong orientation, thereby forcing head-on collisions between replication and transcription. If these collisions slow down replication forks in a specific area in a fraction of cells, this will lead to a steeper gradient of the replication profile in this area when compared to the wild-type profile (cf. Figure 2 panels i and iii). We found similar deviations at *rrnD* and *rrnG*, but these are less pronounced, in line with encounters being co-directional. However, the fact that deviations can be observed suggests that even co-directional collision events in highly transcribed areas might be problematic to ongoing replication, as recently suggested for *B. subtilis* (20,43). We also noted a deviation of the replication profile in *oriC^+^ oriZ* cells in the area of the *rrnCABE* operon cluster, which also would be in line with this idea (Figure 2ii).

**Figure 4.**
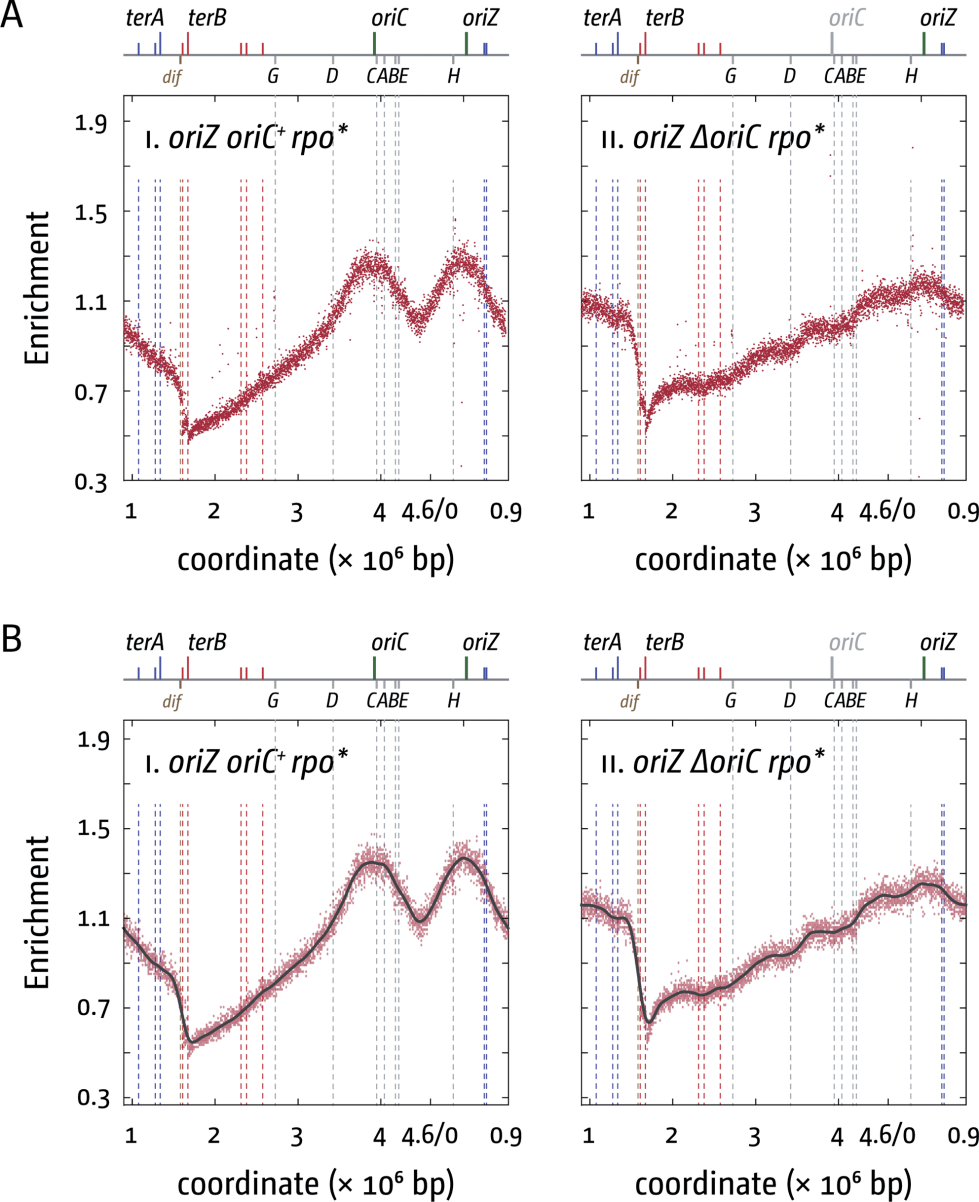
Replication profiles of E. coli rpo* cells with two origins or one ectopic replication origin. (**A**) Marker frequency analysis of *E. coli oriC^+^ oriZ* and *ΔoriC oriZ* cells in which an rpoB*35 point mutation was introduced, which destabilises ternary RNA polymerase complexes. The number of reads (normalised against reads for a stationary phase wild-type control) is plotted against the chromosomal location, starting at 0.9 Mbp for a better visualisation of both replication origins. A schematic representation of the *E. coli* chromosome showing positions of *oriC* and *oriZ* (green line; gray if deleted/not present) and *ter* sites (above) as well as *dif* and *rrn* operons A–E, G and H (below) is shown above the plotted data. The strains used were RCe566 (oriC^+^ oriZ rpo*) and RCe573 (ΔoriC oriZ rpo*). (**B**) Panels i and ii show a LOESS regression curve (see ‘Material and Methods’ section) of the marker frequency data. As the LOESS regression is sensitive to outliers, we removed data points from the sets shown in (A) if they were beyond the threshold (measured enrichment − LOESS value)^2^ > 0.02.

If the deviations are a result of slower replication fork progression caused by head-on replication-transcription collisions an rpoB*35 point mutation (called rpo* from hereon) would be expected to result in a reduction of this effect. This point mutation in RNA polymerase (RNAP) reduces the ability of transcribing RNAP complexes to pause and backtrack (18), thereby alleviating conflicts between replication and transcription (18,19). This was indeed observed. In oriC^+^ oriZ rpo* cells we observed that the deviation at the *rrnCABE* operon cluster disappeared (Figure 4i; see Supplementary Figure S2 for a replication profile of an rpo* single mutant), which supports the idea that replication-transcription encounters in highly transcribed areas are problematic even if they are co-directional (20,43). In ΔoriC oriZ rpo* cells the deviations of the replication profile at all *rrn* operons were still observed, but they were noticeably reduced (Figure 4ii; Supplementary Figure S3), in line with the idea that the problems of head-on replication-transcription conflicts are partially alleviated. This is also reflected in the doubling times. Introduction of an rpo* point mutation into *oriC^+^ oriZ* cells resulted only in a moderately elongated doubling time (Table 2), as observed for rpo* single mutants (Table 2; Supplementary Figure S1B). However, the doubling time of ΔoriC oriZ rpo* cells is significantly quicker than the doubling time of *ΔoriC oriZ* cells (Table 2), especially if the delay caused by rpo* in wild-type cells (Table 2; Supplementary Figure S1B) is taken into consideration.

**Figure 5.**
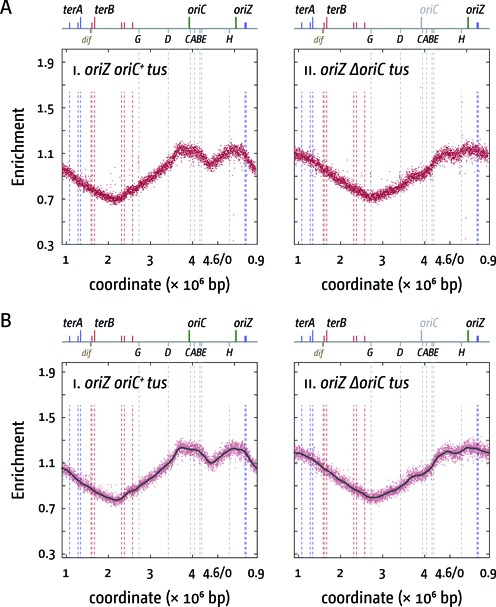
Replication profiles of *E. coli tus* cells with two origins or one ectopic replication origin. (**A**) Marker frequency analysis of *E. coli oriC^+^ oriZ* and *ΔoriC oriZ* cells in which the replication fork trap in the termination area was inactivated by deletion of the *tus* gene. The number of reads (normalised against reads for a stationary phase wild-type control) is plotted against the chromosomal location, starting at 0.9 Mbp for a better visualisation of both replication origins. A schematic representation of the *E. coli* chromosome showing positions of *oriC* and *oriZ* (green line; gray if deleted/not present) and *ter* sites (above) as well as *dif* and *rrn* operons A–E, G and H (below) is shown above the plotted data. The strains used were RCe567 (*oriC^+^ oriZ tus*) and RCe572 (*ΔoriC oriZ tus*). (**B**) Panels i and ii show a LOESS regression curve (see ‘Material and Methods’ section) of the marker frequency data. As the LOESS regression is sensitive to outliers, we removed data points from the sets shown in (A) if they were beyond the threshold (measured enrichment − LOESS value)^2^ > 0.02.

Given that the replication fork trap enforces the different lengths of the replichores in *oriZ* cells, any form of inacti­vation of the Tus terminator protein or *ter*/Tus interaction would at least partially alleviate the asymmetry of the replichores. To investigate whether this was the case we crossed a *tus^−^* allele into the *oriC^+^ oriZ* background. The doubling time of an *oriC^+^ oriZ tus* construct was not significantly different (Table 2). However, as shown in Figure 5, the replication profile changed rather dramatically (cf. Figures 2ii and 5i). The ‘step’ in the termination area of *oriC^+^ oriZ* cells disappeared in the *tus* derivative, resulting in an almost perfectly symmetrical replication profile where forks terminate at 2.199 Mbp, in close proximity to the calculated mid-point between *oriZ* and *oriC* at 2.1335 Mbp (Figure 3; Table 3). *ΔoriC oriZ* cells with a *tus* deletion showed a doubling time of 29.2 min, which is indeed significantly shorter than the doubling time of a *ΔoriC oriZ* construct (Table 2). This effect was supported by the observed replication profile. While the replication profile showed the deviations at *rrnH* and *rrnCABE, ΔoriC oriZ tus* cells showed a termination point that was moved even further, with the minimum of the LOESS curve being located at 2.769 Mbp. This termination point is skewed, as the calculated termination point would be located at 2.664, ∼100 kb closer to the termination area (Figure 3; Table 3). It is likely that the head-on replication-transcription encounters at *rrn* operons H, C, A, B and E contribute toward the observed asymmetry.

**Figure 6.**
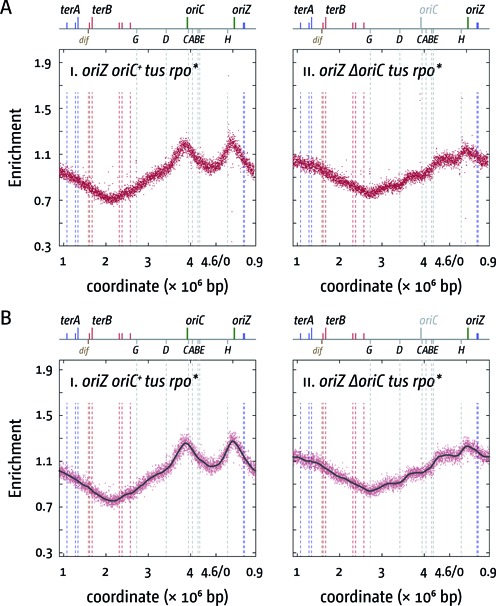
Replication profiles of E. coli tus rpo* cells with two origins or one ectopic replication origin. (**A**) Marker frequency analysis of *E. coli oriC^+^ oriZ* and *ΔoriC oriZ* cells in which the replication fork trap in the termination area was inactivated by deletion of the *tus* gene and an rpoB*35 point mutation was introduced to destabilise ternary RNA polymerase complexes. The number of reads (normalised against reads for a stationary phase wild-type control) is plotted against the chromosomal location, starting at 0.9 Mbp for a better visualisation of both replication origins. A schematic representation of the *E. coli* chromosome showing positions of *oriC* and *oriZ* (green line; gray if deleted/not present) and *ter* sites (above) as well as *dif* and *rrn* operons A–E, G and H (below) is shown above the plotted data. The strains used were RCe574 (oriC^+^ oriZ tus rpo*) and RCe576 (ΔoriC oriZ tus rpo*). (**B**) Panels i and ii show a LOESS regression curve (see ‘Material and Methods’ section) of the marker frequency data. As the LOESS regression is sensitive to outliers, we removed data points from the sets shown in (A) if they were beyond the threshold (measured enrichment − LOESS value)^2^ > 0.02.

This was confirmed by combining an rpo* point mutation with the deletion of *tus* in *oriC^+^ oriZ* and *ΔoriC oriZ* backgrounds. oriC^+^ oriZ tus rpo* cells had a doubling time only marginally slower than oriC^+^ oriZ rpo* cells (Table 2). However, given that both *tus* and rpo* separately improve growth of *ΔoriC oriZ* cells quite substantially, we were surprised to find the doubling time of the ΔoriC oriZ tus rpo* construct to be very similar to the doubling times of *ΔoriC oriZ tus* and ΔoriC oriZ rpo* cells (Table 2). Given that an rpo* point mutation slows growth mildly (Table 2; Supplementary Figure S1B) it is hard to deduce whether the combination of both mutations has a significant effect. The analysis of the replication profiles revealed a ‘blending’ of the properties of both single mutants. oriC^+^ oriZ tus rpo* cells showed a mild shift of the main termination point closer towards the calculated termination point (Table 3; Figure 6), in line with a facilitated progression of forks through *rrnD* and *rrnG*. In addition, the skew of the profile observed at the *rrnCABE* operon cluster in *oriC^+^ oriZ* and *oriC^+^ oriZ tus* cells has disappeared, as observed before for the oriC^+^ oriZ rpo* profile. Upon deletion of *oriC*, the profile shows another shift of the main termination point to 2.709 Mbp. As noted for oriC^+^ oriZ tus rpo* cells, the termination point in ΔoriC oriZ tus rpo* moves even closer to the theoretical mid point (2.664 Mbp) in comparison to *ΔoriC oriZ tus* cells, which is in line with a facilitated progression of forks through highly transcribed chromosomal areas by the rpo* point mutation. However, as observed for the other oriZ rpo* constructs, ΔoriC oriZ tus rpo* cells show deviations of the profile at the locations of all *rrn* operons, regardless of their orientation.

**Figure 7.**
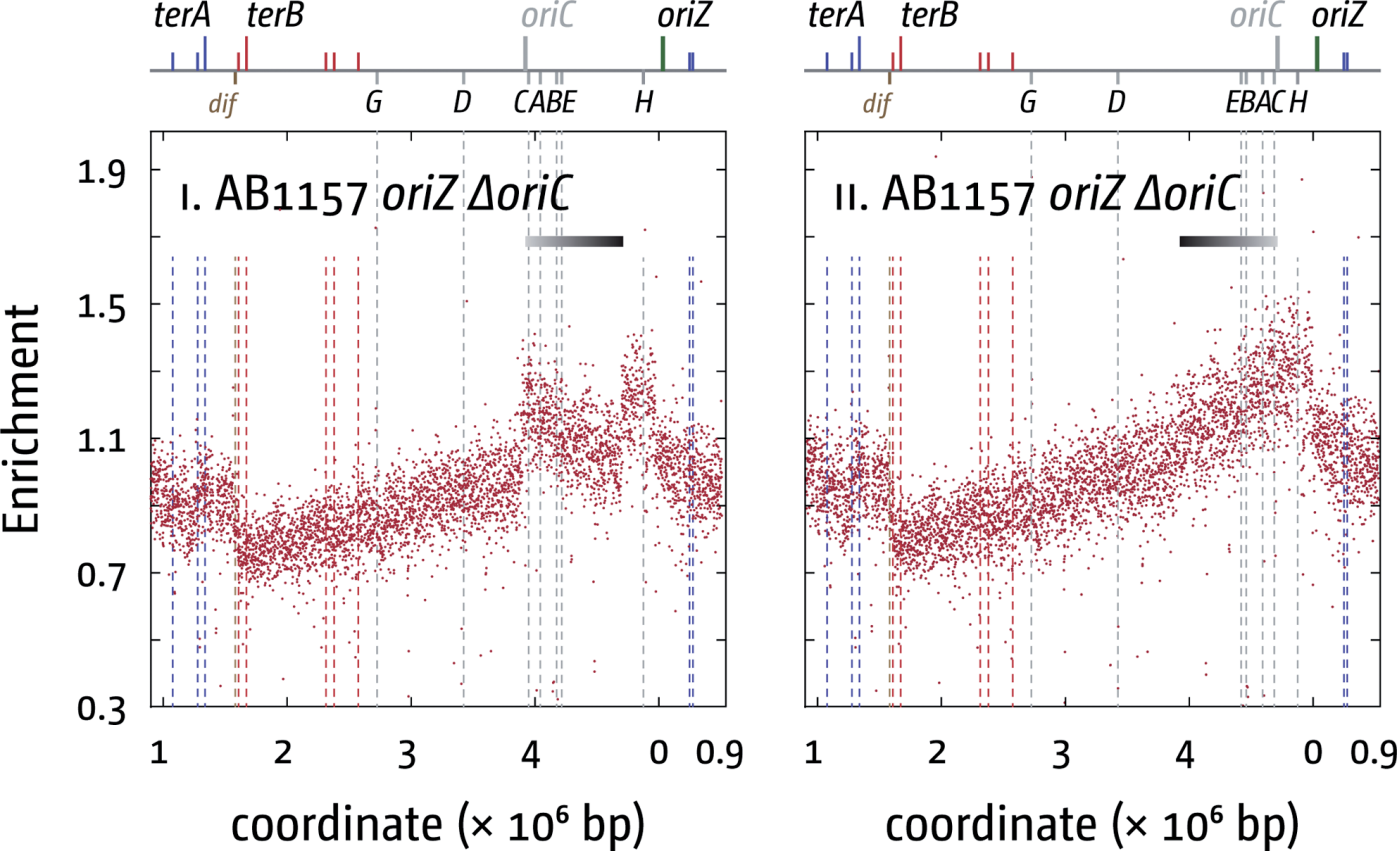
Replication profiles of a fast growing *E. coli* AB1157 *ΔoriC oriZ* derivative (26). The number of reads (normalised against reads for a stationary phase wild-type control) is plotted against the chromosomal location, starting at 0.9 Mbp for a better visualisation of both replication origins. A schematic representation of the *E. coli* chromosome showing positions of *oriC* and *oriZ* (green line; gray if deleted/not present) and *ter* sites (above) as well as *dif* and *rrn* operons A–E, G and H (below) is shown above the plotted data. The clear deviation from the host strain profile indicates an inversion (38) (highlighted by the bar above with a black to gray gradient), spanning from the deleted *oriC* area to the *leuABC* genes at 0.082 Mbp. In panel ii, this section was inverted to demonstrate the continuity of the profile. The drop in the marker frequency just downstream of the inserted *oriZ* is likely to be caused by the presence of an array of 240 copies of the *tetO* operator, which acts as a block to replication. The strain used for extraction of chromosomal DNA was WX340 (AB1157 *ΔoriC oriZ*).

Thus, both the inactivation of the replication fork trap as well as a facilitated progression of forks through highly transcribed areas partially suppress the growth defect observed in *ΔoriC oriZ* cells and we observed that the formation of spontaneous point mutations appears much reduced in ΔoriC oriZ rpo*, ΔoriC oriZ tus and ΔoriC oriZ tus rpo* cells (Supplementary Figure S4). To identify the compensatory mutation which might contribute to the quick replication time of the previously published AB1157 *ΔoriC oriZ* construct (26), we established a replication profile of this strain to identify which suppression mechanism is allowing the quick replication time. The profile shown in Figure 7 shows a stunningly simple reason for the quick doubling time. The strain carries a gross chromosomal rearrangement, which roughly spans from the deleted *oriC* region (3.920 Mbp) to the *leuABC* area (0.082 Mbp) (see Supplementary Figure S5 for details). This inversion leaves the region in between *leuABC* and *oriZ* (0.334 Mbp), which includes *rrnH*, intact, which will trigger head-on collisions between replication and transcription in this ∼250 kb stretch of the chromosome. However, the remaining portion of the chromosome (*leu–ΔoriC*) is inverted, including the *rrnCABE* operon cluster. Thus, replication-transcription encounters within this 800 kb stretch in cells replicating from *oriZ* only will be as they would have been in wild-type cells with replication initiating at *oriC*. This provides a surprisingly simple explanation why this strain has such a short doubling time. As with our *ΔoriC oriZ* construct, one replichore is indeed significantly longer, but the majority of issues arising from replication-transcription conflicts are simply eliminated, resulting in a much quicker doubling time. The fact that such a gross chromosomal rearrangement was purified illustrates the severity of the impact of head-on collisions between replication and transcription on ongoing chromosomal replication.

## DISCUSSION

The *E. coli* chromosome shows distinct organisational features. It is replicated from a single replication origin. In addition, the specialised termination area acts as a replication fork trap, allowing forks to enter but not to leave. This set-up ensures that each replichore is replicated by one replication fork only. Thus, replication in each replichore has a distinct direction and forks are actively prevented from entering the opposite replichore. To some extent the same is observed for the directionality of transcription, not only in *E. coli*, but in many bacterial species. In *E. coli* the overall co-directionality of transcription and replication is only 54% (11), but >90% of genes coding for ribosomal proteins are transcribed co-directionally with DNA replication (10,11). The co-directionality of replication and transcription is higher in other bacteria, with *B. subtilis* showing a general co-directionality of replication and transcription of almost 75% (11). The overall co-directionality of replication and transcription, especially in terms of highly transcribed genes, has led to the hypothesis that head-on replication-transcription encounters might be problematic (10,12,18,20), an idea supported by *B. subtilis* experiments in which the origin was moved to an ectopic location (24,25) as well as experiments in *E. coli* strains with inverted *rrn* operons (21,22). In the light of these results it was a surprise that *E. coli* strains with an asymmetric replication profile forced by an ectopic replication origin did not lead to a specific delay of chromosomal replication (26).

In this study, we have generated a similar construct. Our data contrast the previous study and confirm that cells with an ectopic replication origin show a severe delay of the duplication time, which is at least twice that of normal growing *E. coli* cells (Table 2). The replication profile of a *ΔoriC oriZ* construct revealed *terC* and *terB* as locations where the vast majority of forks replicating the shorter replichore are stopped, in line with previous reports indicating that *ter*/Tus are stable replication fork-arresting complexes (44). Thus, our data confirm that replication in a *ΔoriC oriZ* construct is asymmetric, with one fork having to replicate approx. 3.3 Mbp, while the other replicates approx. 1.3 Mbp (Figures 2 and 3) and there is no indication that forks from the shorter replichore enter the other replichore with a high frequency.

This asymmetry is mostly resolved upon deletion of *tus* (Figures 3 and 5). Whilst the deletion of *tus* has little effect on the replication profile in wild-type cells (Figure 3) (41), the termination point in *oriC^+^ oriZ tus* cells is shifted and the LOESS minimum is observed 70 kb away from the calculated midpoint between initiation sites. Similarly, in *ΔoriC oriZ tus* cells the termination point is shifted even further and replication terminates roughly 100 kb away from the calculated mid-point (Figures 3 and 5). Overall, the profiles of *tus* cells, regardless of whether they are replicated from two origins or *oriZ* only, show few deviations in comparison to the profile of wild-type cells, which would be indicative of problems in the area where forks proceed beyond the *ter*/Tus boundaries, thereby moving in an orientation opposite to normal. In fact, the clockwise *oriZ*-replichore, whose fork will proceed past the termination area into the opposite replichore, is the longer replichore, suggesting that on average fork speed of the fork going partially against transcription is greater than the fork coming from *oriC* travelling in the correct orientation. In contrast, the anti-clockwise *oriZ* replichore shows a number of areas indicative of replication problems (Figure 1). The most noticeable deviations coincide with *rrnH* as well as the *rrnCABE* operon cluster. We cannot exclude that the deviations observed are partially caused by technical artefacts such as persistent protein-DNA interactions, which might bias extraction of genomic DNA. However, these deviations, if caused specifically by technical artefacts, should be observed in all strains, which is not the case. They are specifically observed if replication is going against the directionality of transcription and, in addition, they are partially alleviated if the stability of RNA polymerase is reduced, suggesting the observed effects must be more than just a technical artefact. We prefer the interpretation that these strong deviations at *rrnH* and the *rrnCABE* cluster in *ΔoriC oriZ* and *ΔoriC oriZ tus* cells highlight that head-on replication of specifically these highly transcribed areas causes substantial problems to replication progression (Figures 2 and 5), in line with the observations in *B. subtilis* (24,25).

The idea that head-on collisions of replication and transcription specifically at the *rrnH* and *rrnCABE* areas cause delays to fork progression (Figures 2 and 5) is supported by our finding that the deviations observed are reduced if an rpo* point mutation is introduced into these backgrounds (Supplementary Figure S3). While the doubling time of oriC^+^ oriZ rpo* cells is slightly elongated in comparison to *oriC^+^ oriZ* cells, an rpo* point mutation significantly improves the doubling time of *ΔoriC oriZ* constructs, in line with the idea that replication fork progression through *rrnH* and the *rrnCABE* cluster is facilitated. Indeed, in both *oriC^+^ oriZ tus* and *ΔoriC oriZ tus* cells the addition of an rpo* point mutation results in a shift of the termination point towards the calculated mid-point, indicating that the overall speed of replication of both replichores becomes more even (cf. Figures 2, 4 and 5).

However, the nature of the suppressor mutation that has allowed the previously reported AB1157 *ΔoriC oriZ* to grow with a doubling time close to wild-type cells (26) provides the strongest supporting argument for the hypothesis that highly transcribed genes and especially the *rrnCABE* cluster are a major obstacle to replication. It shows that an 800 kb inversion, which includes specifically *rrnCABE*, but not *rrnH*, mostly alleviates the growth defect observed if the origin is moved to an ectopic location. Thus, head-on collisions of replication forks and transcribing RNA polymerase complexes at the *rrnCABE* cluster appear to contribute most to the growth defect observed.

We noted a deviation of the replication profile at the *rrnCABE* operon cluster even if it was replicated co-directionally with transcription (Figures 2 and 5), an effect that disappeared if an rpo* point mutation was introduced (Figures 4 and 6). It was recently observed in *B. subtilis* that replication-transcription encounters can cause a problem even if they are co-directional (20,43), which makes it tempting to speculate that the observed deviation might be caused by a similar effect. However, we did not observe a similar distortion in our wild-type profile (Figure 2). One potential explanation for this discrepancy might be the fact that in normal cells the total number of replisomes is relatively low (on average ∼10 per cell (45)). In cells with two origins an increased number of replisomes will be in use, as both origins initiate synthesis simultaneously, thereby causing an overall reduction of available replisome components. Thus, if forks occasionally are stalled in an *rrn* operon, restart might be delayed, as replisome components might become limiting. However, we did not observe these deviations when we previously sequenced an AB1157 *oriC^+^ oriZ* derivative (41). Thus, it remains to be established how much co-directional replication-transcription encounters impede replication fork progression in *E. coli*.

We were interested to find that, upon *ter*/Tus inactivation, replication appears to proceed with ease into the opposite replichore. This opens the question as to whether the prevention of forks of one replichore entering the other in the wrong orientation is an important purpose of the replication fork trap (10,13,19). The majority of highly transcribed genes and especially the *rrn* operons are located in relative proximity to the origin with few exceptions (Figure 1A) (10), making it less likely that a fork escaping from the termination area will ever enter them from the wrong side. In line with this we recently published a series of results offering an additional explanation. We demonstrated that RecG helicase is a key player for processing intermediates arising from the collision of two replication forks. Our genetics and cell biology data is in line with the idea that the collision of two approaching replisomes might result in the formation of a 3′ flap structure. This 3′ flap, while normally processed by RecG or 3′ exonucleases, persists in *recG* cells and is processed instead by PriA and recombination proteins, triggering the recruitment of additional replication forks which start to over-replicate an already replicated area (41). This over-replication is efficiently contained by the *ter*/Tus replication fork trap. Indeed, it was shown that in *tus* cells a low but detectable level of over-replication was observed (46), indicating that even in the presence of RecG and 3′ exonucleases replication fork fusion might generate potentially harmful intermediates, which can be contained as long as the replication fork trap is active. Thus, the dangers associated with replication fork fusion might not only provide a potential explanation for the importance for a replication fork trap in the termination area, but it might also explain why bacterial chromosome are replicated from a single replication origin, thereby limiting the number of fork fusions. A chromosomal architecture with one defined replication origin and a distinct termination area, which results in two defined replichores, allows an uncomplicated way to not only coordinate co-directionality of replication and transcription, but also minimises the number of replication fork collision events to exactly one per cell cycle.

## ACCESSION NUMBERS

All relevant raw sequencing data can be accessed at the European Nucleotide Archive (http://www.ebi.ac.uk/ena/data/view/PRJEB9476).

## SUPPLEMENTARY DATA

Supplementary Data are available at NAR Online.

SUPPLEMENTARY DATA
