# Supplementary material for: Shaping the landscape of the Escherichia coli chromosome: replication-transcription encounters in cells with an ectopic replication origin
Source: Nucleic Acids Res. 2015 Jul 8;43(16):7865–77. doi: 10.1093/nar/gkv704 (PMC4652752; doi:10.1093/nar/gkv704)
Supplement: SUPPLEMENTARY DATA [file supp_43_16_7865__index.html]

Shaping the landscape of the Escherichia coli chromosome: replication-transcription encounters in cells with an ectopic replication origin — Shaping the landscape of the Escherichia coli chromosome: replication-transcription encounters in cells with an ectopic replication origin — SUPPLEMENTARY DATA 

# Shaping the landscape of the *Escherichia coli* chromosome: replication-transcription encounters in cells with an ectopic replication origin

## SUPPLEMENTARY DATA

- SUPPLEMENTARY DATA
