# Supplementary material for: Shaping the landscape of the Escherichia coli chromosome: replication-transcription encounters in cells with an ectopic replication origin
Source: Nucleic Acids Res. 2015 Jul 8;43(16):7865–77. doi: 10.1093/nar/gkv704 (PMC4652752; doi:10.1093/nar/gkv704)
Supplement: SUPPLEMENTARY DATA [file supp_gkv704_nar-01507-v-2015-File009.pdf]

Shaping the landscape of the *Escherichia coli* chromosome: replication-transcription encounters in cells with an ectopic replication origin

Supplementary information

Darja Ivanova, Toni Taylor, Sarah L. Smith, Juachi U. Dimude, Amy L. Upton, Mana M. Mehrjouy, Ole Skovgaard, David J. Sherratt, Renata Retkute and Christian J. Rudolph

Supplementary Material & Methods

Growth curves

The growth curves of MG1655 and *rpoB*35* in Figure S1B were recorded before, using a slightly different experimental procedure (1). The strains indicated were grown in LB broth to an A_{650} of 0.2. The culture was diluted 10,000-fold in conditioned medium, which was created by growing the wild-type strain in fresh LB broth to an A_{650} of 0.2 with subsequent sterile filtration. The diluted cells were incubated in a 37 °C shaking water bath and at each time point samples were removed, mixed with 2.5 mL of molten 0.6 % top agar kept at 42 °C, and plated on LB agar. At later time points, the samples were diluted a further 10- or 100-fold in conditioned medium before plating. Colonies were counted after incubation for 18 – 24 h at 37 °C.

Supplementary References

1. Rudolph,C.J., Upton,A.L. and Lloyd,R.G. (2007) Replication fork stalling and cell cycle arrest in UV-irradiated *Escherichia coli.* Genes Dev., 21, 668 – 681.
2. Rudolph,C.J., Upton,A.L., Stockum,A., Nieduszynski,C.A. and Lloyd,R.G. (2013) Avoiding chromosome pathology when replication forks collide. Nature, 500, 608 – 611.
3. Wang,X., Lesterlin,C., Reyes-Lamothe,R., Ball,G. and Sherratt,D.J. (2011) Replication and segregation of an *Escherichia coli* chromosome with two replication origins. Proc. Natl. Acad. Sci. U. S. A., 108, E243 – 250.

Darja Ivanova, Toni Taylor, Sarah L. Smith, Juachi U. Dimude, Amy L. Upton, Mana M. Mehrjouy, Ole Skovgaard, David J. Sherratt, Renata Retkute and Christian J. Rudolph

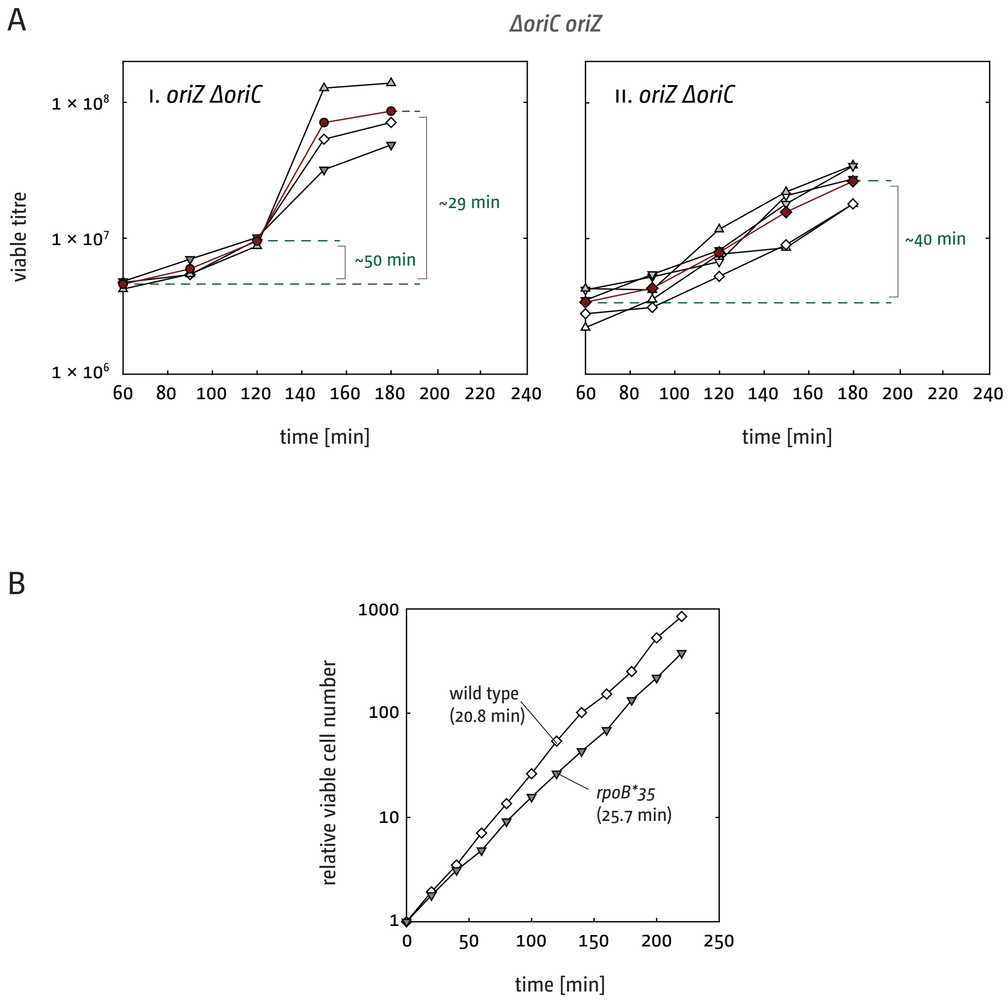

Supplementary Figure 1. Cellular replication of *E. coli* strains with one origin in its native or in an ectopic location. **A)** Growth curves of a strain in which the origin was moved to an ectopic location (*ΔoriC oriZ*). In panel i the growth curves were initiated by inoculation from a fresh overnight culture and show a clear biphasic pattern, which is likely to be caused by fast growing cells carrying suppressor mutations and outgrow the slow *ΔoriC oriZ* construct. In panel ii initial inoculation of a starter culture was done directly from a single colony on a streak plate, carefully avoiding larger colonies which might already carry a suppressor mutation. The strain used was RCe578 (*ΔoriC oriZ*). See Material and Methods for details on how the growth curves were recorded. **B)** Growth curves of MG1655 and an *rpoB*35* single mutant. The growth curves of both strains were recorded *via* a slightly different experimental set-up, as detailed in Supplementary Methods. The strains used were MG1655 and N4849 (*rpoB*35*). The data are the mean of two experiments that gave almost identical values.

Darja Ivanova, Toni Taylor, Sarah L. Smith, Juachi U. Dimude, Amy L. Upton, Mana M. Mehrjouy, Ole Skovgaard, David J. Sherratt, Renata Retkute and Christian J. Rudolph

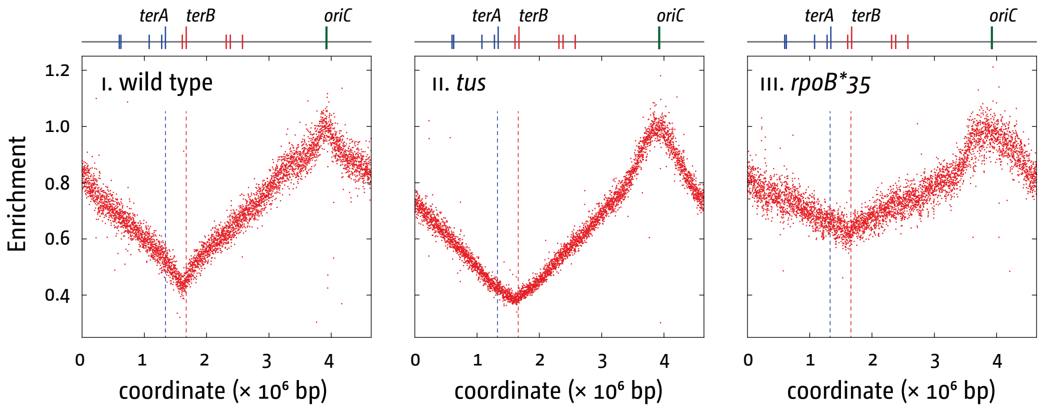

Supplementary Figure 2. Effects of *tus* and *rpo** mutations on chromosome marker frequencies in exponential-phase cells cultured at 37 °C. The data for MG1655, *tus* (N8227) and *rpo** (N5925) were re-plotted from (2).

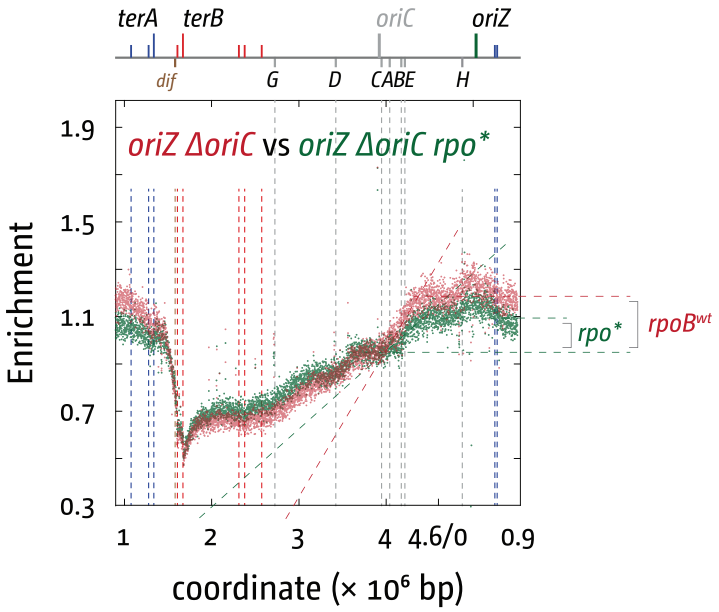

Supplementary Figure 3. Comparison of the replication profiles of *ΔoriC oriZ* and *ΔoriC oriZ rpo** cells. Introduction of an *rpo** point mutation changes the "step" as well as the angle observed at the position of the *rrnCABE* operon cluster, as indicated by dotted lines. The data sets are reproduced from Figures 2 and 4.

Darja Ivanova, Toni Taylor, Sarah L. Smith, Juachi U. Dimude, Amy L. Upton, Mana M. Mehrjouy, Ole Skovgaard, David J. Sherratt, Renata Retkute and Christian J. Rudolph

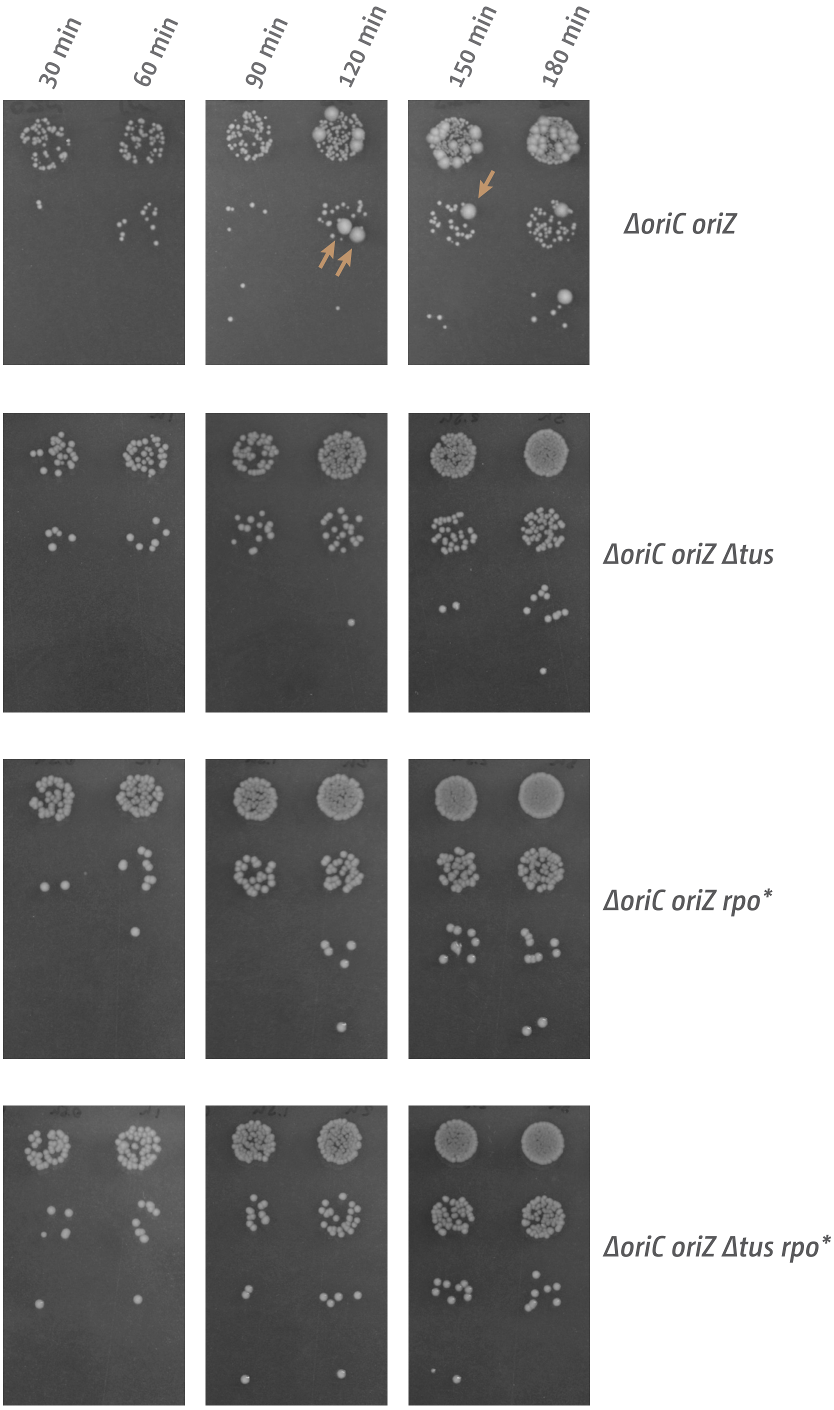

Supplementary Figure 4. Growth and suppressor formation in strains lacking *oriC*. Evaluation of the viable titre of *ΔoriC oriZ rpo**, *ΔoriC oriZ tus* and *ΔoriC oriZ tus rpo** cells via spot dilutions of a growing culture at the times indicated. Some large colonies within the small *ΔoriC oriZ* colonies which have developed suppressor mutations are highlighted by brown arrows. The strains used was RCe572 (*ΔoriC oriZ tus*), RCe573 (*ΔoriC oriZ rpo**) and RCe576 (*ΔoriC oriZ tus rpo**). RCe578 (*ΔoriC oriZ*) was reproduced from Figure 1 for comparison.

Junction 1 – *oriC* region (3.92 Mbp)

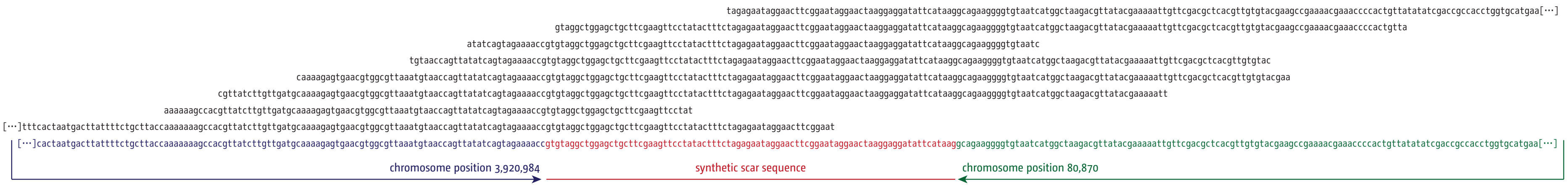

Supplementary Figure 5. Consensus sequence and a selection of sequence reads covering one of the two junctions of the gross chromosomal rearrangement. A representative selection of reads from the Deep Sequencing data is shown to demonstrate continuous coverage of the junction near *oriC* of the chromosomal inversion. The sequence highlighted in blue is from the 3.9209 Mbp region of the chromosome near *oriC*, whereas the sequence highlighted in green is from the 0.0808 Mbp region near *leuA*. The sequences are joined by a scar sequence (red) which is synthetic and stems from previous manipulations of the chromosome (see strain table and (3)). The second junction is complex due to prior chromosome manipulations and is not shown.
